# Management of late-onset deep surgical site infection after instrumented spinal surgery

**DOI:** 10.1186/s12893-018-0458-4

**Published:** 2018-12-22

**Authors:** Dong Yin, Bin Liu, Yunbing Chang, Honglin Gu, Xiaoqing Zheng

**Affiliations:** Department of Orthopaedics, Guangdong General Hospital, Guangdong Academy of Medical Sciences, P. O. Box 510080, No.106 Zhongshan Er Road, Yuexiu District, Guangzhou, China

**Keywords:** Late-onset, Surgical site infection, Spinal surgery, Instrumentation, Management

## Abstract

**Background:**

There are no universally accepted protocols for the treatment of late-onset deep surgical site infection. This study retrospectively evaluates the methods of aggressive debridement with instrumentation retention, high vacuum closed-suction drain without irrigation, primary wound closure, and antibiotic therapy for the treatment of late-onset deep surgical site infection after instrumented spinal surgery.

**Methods:**

A total of 4057 patients who underwent instrumented spinal surgeries were surveyed from January 2010 to June 2014. Surgical debridement was performed immediately after late-onset deep surgical site infection was identified. In addition to extended resection of the devitalized and necrotic tissue, the biofilms adhered to the surface of implants were removed meticulously and thoroughly. Primary wound closure was performed on each patient, and closed suction drains were maintained for about 7–10 days without irrigation. Antibiotic therapy was administered for 3 months according to the results of the pathogenic culture.

**Results:**

Forty-two patients were identified as having late-onset deep surgical site infection. *Staphylococcus aureus* was the most common pathogen in this group. Seven patients with late-onset deep surgical site infection had negative bacterial culture results. Infections resolved in all patients. Forty-one patients retained their instrumentation, whereas 1 patient had the implants removed because of *Staphylococcus aureus* infection, which was found the implants loosening during debridement. Primary wound healing was found in all patients with no recurrence of infection during the follow-up periods.

**Conclusions:**

A timely diagnosis, aggressive and meticulous debridement, high vacuum closed-suction drain, routine and adequate use of antibacterial agents are the keys to successfully resolving infection and keeping implants retention in the treatment of late-onset deep surgical site infection after instrumented spinal surgery.

## Background

Although basic measures for decreasing postoperative surgical site infection (SSI) have been emphasized and considered, especially with instrumentation, late-onset deep SSI is still one of the most complicated problems in spinal surgery. It results in long-term antibiotic use, re-operation, and prolonged hospitalization, and it remains among the leading causes of morbidity and mortality. The incidence of SSI after instrumented spinal surgery has been reported to range from 2.2 to 20% [1–6]. Regarding the treatment of late-onset deep SSI after spinal instrumentation, most studies have suggested that it requires complete removal of the implants [1, 7–9]. Implant retention may prevent bacteria eradication because of the presence of biofilms on metal instrumentation, which diminishes the effect of antibiotics. However, implant removal before graft fusion for attempted infection control can be problematic because spinal instability may result, causing clinical symptoms, such as backache, radicular pain, or neurologic deficits. Thus, those two aims are often contradictory. Is it possible to resolve the infection as well as to keep implant retention? The objective of the present study is to evaluate the methods of aggressive debridement with instrumentation retention, high vacuum closed-suction drain without irrigation, primary wound closure, and antibiotic therapy for the treatment of late-onset deep SSI after instrumented spinal surgery.

## Methods

A retrospective survey of 4057 patients who underwent dorsal spinal surgeries with instrumentation was conducted in the spine center of our institution between January 2010 and June 2014. The diagnosis of late-onset deep SSI was confirmed by symptoms such as persistent back pain, signs such as wound inflammation with fluctuation or discharge, histopathological findings, radiologic examination (magnetic resonance imaging, MRI), and laboratory findings, including a complete blood count, erythrocyte sedimentation rate (ESR), C-reactive protein (CRP), and procalcitonin (PCT). Sometimes color Doppler ultrasonography was used to identify a condition resulting in an abscess or other evidence of infection in the deep soft tissue, muscle, and fascia.

**Fig. 1:**
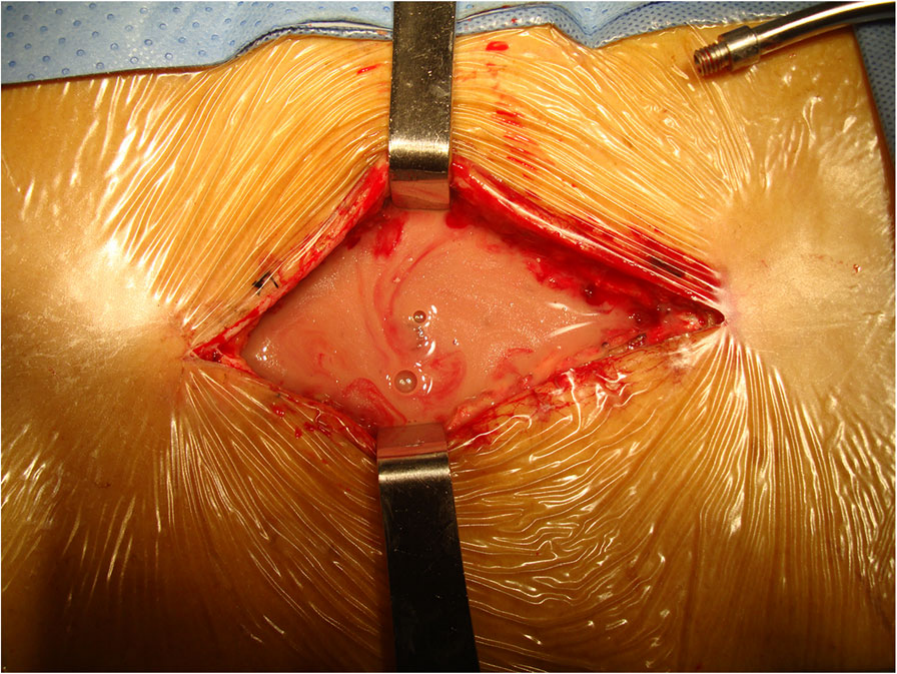
Pus deep in the wound was completely removed using a sucker

**Fig. 2:**
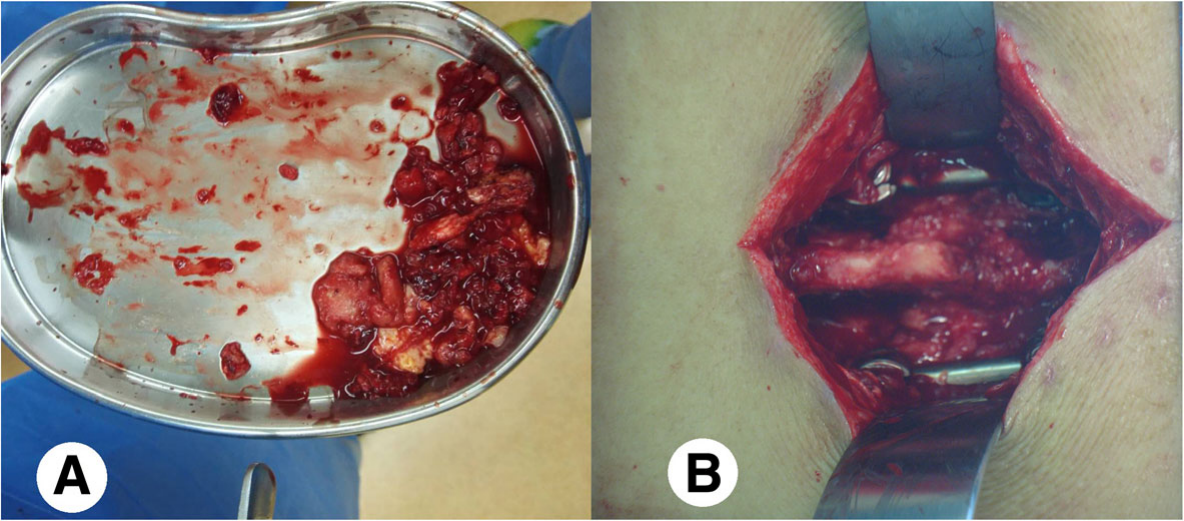
**a** Devitalized and necrotic tissue was extensively resected, including the cyst wall around the abscess; **b** The biofilms adherent to the surface of the implants were eliminated thoroughly

**Fig. 3:**
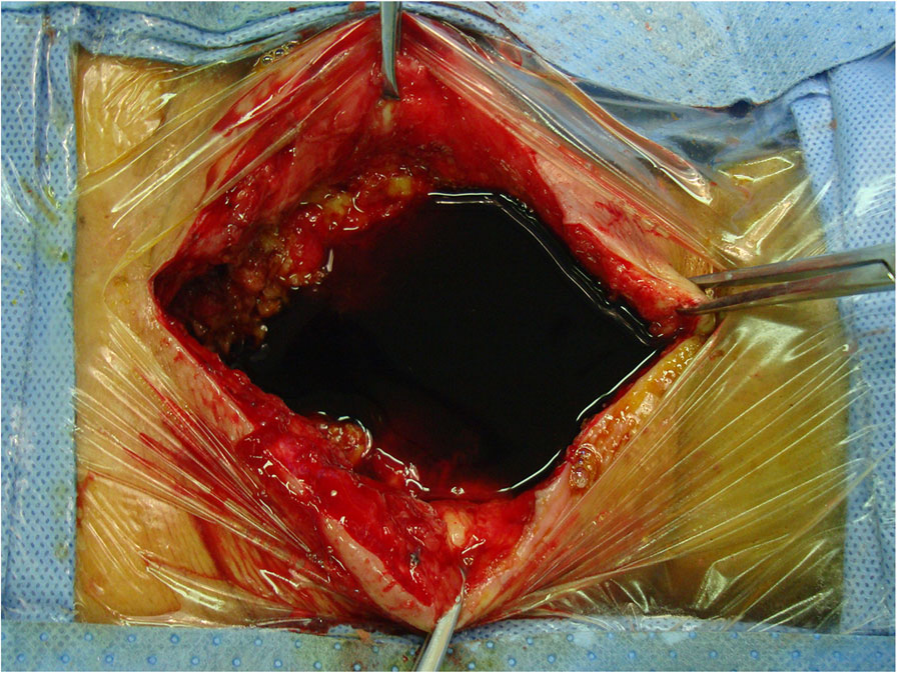
The wound was soaked in povidone-iodine solution for 5 to 10 min.

Aggressive, meticulous surgical debridement of all devitalized tissue was performed on every infected patient in a timely manner. The incision was made using a posterior approach, i.e., the same approach used during the initial surgery. Firstly, the pus was completely removed using a sucker (Fig. 1). Secondly, the devitalized and necrotic tissue was extensively resected, including the cyst wall around the abscess (Fig. 2a). The biofilms adhered to the surface of the implants were eliminated thoroughly (Fig. 2b). Bacterial cultures and drug sensitivity tests of the necrotic tissue and biofilm were performed to prescribe accurate postoperative antibiotic therapy. Then the wound was washed using disinfectants in the following sequence: hydrogen peroxide solution, normal saline, povidone-iodine solution, and normal saline again. The wounds were usually soaked with povidone-iodine solution for 5–10 min (Fig. 3). After these procedures, primary wound closure was then performed. The fascia was closed tightly with both interrupted and running sutures, and the subcutaneous tissues and skin were closed routinely, followed by the placement of 2 closed-suction drains that were maintained for 7 to 10 days.

Based on bacterial culture and drug sensitivity test, intravenous antibiotics were administered for a minimum of 6 weeks, followed by a 6-week course of oral antibiotic therapy in each infected patient. The total period of antibiotic therapy was at least 3 months. The mean follow-up period was 46.64 months (range, 24 to 72 months).

## Results

Forty-two patients (1.04%) were identified as having late-onset deep SSIs. There were 25 men and 17 women who had a mean age of 68.64 years (range, 53 to 83 years). Late-onset deep SSIs were occurred in the 22.19 weeks postoperatively (range, 6 weeks to 1 year). Among the patients suffering late-onset deep SSI, 3 patients (7.14%) underwent laminoplasty of the cervical spine, and 39 (92.86%) underwent transforaminal lumbar interbody fusion (TLIF) or posterior lumbar interbody fusion (PLIF) of the lumbar spine. Details about patients’ data, including preoperative American Society of Anesthesiologists (ASA) physical status and presence of diabetes mellitus, are listed in Table 1.

**Table 1:** Background data and details of patients with delayed deep SSI

		Cervical spine	Lumbar spine
Number		3	39
Age		65.67	68.87
Gender (M:F)		2:1	23:16
ASA physical status	1	2	13
	2	1	24
	3		2
No. of patients with diabetes mellitus			11
No. of fused segments			50
Laminoplasty		3	
PLIF			26
TLIF			13

The infectious organisms are shown in Table 2. *Staphylococcus aureus* was the most common pathogen of late-onset deep SSI in this group, and the second most common pathogen was *Escherichia coli*. Seven patients with late-onset deep SSI had negative bacterial culture results. Forty-one patients retained their instrumentation, whereas 1 patient had the internal fixation device removed because of a *Staphylococcus aureus* infection. This 72-year-old man underwent posterior lumbar interbody fusion (L4/5). He experienced low back pain and had signs of wound inflammation with fluctuation 13 weeks postoperatively. The laboratory examination showed increasing serum ESR, CRP, and PCT levels. The MRI examination showed a high-intensity signal in the deep tissue. Surgical debridement was performed immediately after the diagnosis of deep SSI. Instrumentation was found to be loose upon debridement, and the screws and rods were removed. Nevertheless, infections resolved in all 42 patients in this group with no recurrence during the follow-up period. Primary healing of the surgical incision took place in all cases, and stitches were removed 2 to 3 weeks after debridement.

**Table 2:** Bacteria isolated from intraoperative tissue samples in 42 patients

*Staphylococcus aureus*	13
*Escherichia coli*	7
*ESBL of Escherichia coli*	3
*Enterobacter cloacae*	3
*MRSA*	2
*Acinetobacter baumannii*	2
*Klebsiella pneumoniae*	2
*Enterococcus faecium*	1
*Pseudomonas aeruginosa*	1
*Staphylococcus haemolyticus*	1
No bacteria cultured	7

*ESBL* Extended-spectrum ß-lactamase, *MRSA* Methicillin-resistant *Staphylococcus aureus*

## Discussion

SSI after spinal surgery is an uncommon but disastrous complication. Factors such as age, obesity, diabetes mellitus, smoking, previous surgery, longer duration of surgery, posterior surgical approach, use of spinal instrumentation, poor general or functional status, and the operating room environment are well established in the literature as the risks of infection [2, 5, 6, 10, 11]. Late-onset deep SSI after instrumented spinal surgeries is particularly difficult to manage because of the implanted and possibly infected instrumentation. It is important to know how to treat late-onset deep SSI, especially because it has not been definitively established whether it is best to remove the implants or leave them in place instead. Traditionally, it was thought that spinal instrumentation could act as a culture medium for bacterial growth; therefore, removal of the implants was crucial [1, 7–9]. However, the removal of implants in an attempt at controlling infection before bone-graft fusion may result in loss of correction, spinal instability, and clinical symptoms, such as backache, radicular pain, or neurologic deficits [12]. Infection control and implant retention are often contradictory goals. Nevertheless, current practices vary in terms of the need for implant removal.

### Debridement

Once late-onset deep SSI after instrumented spinal surgery is confirmed, debridement should be performed as early as possible because the infection may spread to nearby areas, which would hinder the treatment of the infection, especially in the case of a high-pressure abscess, and the implants may be loose, leading to internal fixation failure and a lack of adequate spinal fusion. Additionally, certain bacteria can attach to the surface of implants and form a biofilm that makes the infection difficult to treat. The reason for these difficulties is ascribed to the fact that bacteria produce and embed themselves in a matrix of an extracellular polymeric substance and thus can form biofilm colonies that are resistant to antibiotic treatment [13]. Removal of the implants in debridement offers the advantage of eliminating microorganisms harbored in biofilms on the surface of the implants, thus increasing the chance of eradicating the infection. Nevertheless, this potential advantage must be weighed against the risks of prematurely removing implants that are essential for maintaining normal spinal alignment and preserving spinal stability.

Based on the 42 late-onset deep SSI patients, we find that implants can be retained if they are not loosening and if aggressive and meticulous debridement is performed as soon as possible, and the biofilms adhered to the surface of the implants eliminated thoroughly. Washing with hydrogen peroxide and soaking with povidone-iodine solution may be beneficial to primary wound healing.

### Drain placement and irrigation

Although the use of drains in posterior spinal surgery remains controversial [14, 15], a closed suction drain is commonly placed to prevent abscess or epidural hematoma formation, which can lead to spinal cord compression and even paralysis in spinal surgery [16, 17]. Two kinds of drains were placed after debridement in this group: one was a closed-suction drain (CSD) like a Hemovac drain, and the other was a high vacuum wound drainage system (HVWD). HVWD is a useful adjunct that facilitates controlled negative pressure to evacuate wound edema fluid, increase regional blood flow, decrease the bacterial load, and promote the closure of muscle tissue and wound healing, as well as to reduce the risk of retrograde contamination. Vacuum-assisted closure (VAC), a type of device that effectively uses a porous foam sponge to improve wound healing, was not used in this group because primary closure of the wound was performed in all patients. Additionally, severe complications (e.g., uncontrolled sepsis and severe blood loss) have been reported to be associated with the use of VAC [18]. The HVWD was usually maintained for about 7 days postoperatively, and the CSD was removed at about 10 days postoperatively when the discharge was observed to be less than 20 ml.

Wound irrigation has been used to reduce the number of contaminated bacteria in the treatment of SSI. Most authors recommend leaving the wound open after debridement and irrigation with antibiotic normal saline until the wound is sufficiently clean for delayed-staged closure [19, 20]. Although irrigation is effective for treating postoperative wound infections following instrumented spinal fusion, it may cause bacteria to diffuse into the surrounding tissue and make infection control difficult. In this group, primary wound closure was performed in all the 42 patients using HVWD and CSD without any irrigation. The fascia was closed tightly with both interrupted and running sutures. The subcutaneous tissues and skin were closed routinely.

On the basis of the present study, we conclude that it is necessary to place drains after surgical debridement in the treatment of late-onset deep SSI after instrumented spinal surgery, especially a high negative pressure drainage system. Irrigation is not necessary if primary wound closure is performed tightly with a high vacuum closed-suction drainage system after meticulous and thorough debridement.

### Antibiotic therapy

Recommendations regarding the type and duration of antibiotic therapy vary in the literature. Some authors have recommended that antibiotic therapy should be continued for at least 6 weeks intravenously and then for several weeks orally [21, 22]. Other authors have recommended a 2–5 days’ course of intravenous antibiotic administration followed by a 7–14 days’ course of oral antibiotics [9]. However, in the present study, the duration of intravenous antibiotic administration was 6 weeks, followed by oral antibiotic administration for another 6 weeks. The total treatment course of antibiotic therapy was 3 months. Kowalski et al. [8] reported that compared to no suppressive agents, long-term antibiotic therapy was associated with a higher chance (80% vs. 33%) of eradicating infections and retaining implants. Pus, biofilms and necrotic tissue were taken from deep within the wound for bacteria cultures in all the 42 patients. Thirty-five patients (83.33%) had bacterial culture positive results. All isolated bacteria were tested for antibiotic sensitivity, which helped guide an infectious disease specialist in prescribing antibiotic treatment. *Staphylococcus aureus*, which is the most common pathogen involved in skin and soft tissue infections and SSI, was found in 13 patients (30.95%). Late-onset deep site infections are more often culture negative than early infections because they are frequently caused by low virulent pathogens [23, 24]. There were 7 patients (16.67%) who had bacterial culture negative results in this group. It has been suggested that postoperative sterile inflammatory processes may create a favorable environment for the growth of low virulence organisms [25]. Additionally, the period of bacterial culture may have been short or the time from sampling the infected tissue to the laboratory bacterial culture may have been too long, making the pathogen difficult to detect. The rate of positive bacterial culture could be improved if the infected tissue specimens had been placed on the culture medium immediately from the deep wound and the bacterial culture time for proliferation was prolonged. Nevertheless, 6 weeks of intravenous antibiotics followed by 6 weeks of oral antibiotic therapy were also routinely administered in the 7 patients. According to the present study, a routine and adequate use of sensitive antibacterial agents is necessary for infection control as well as implants retention no matter what result of bacterial culture is.

## Conclusions

Instrumentation is now an integral component in the treatment of numerous spinal disorders. The management of late-onset deep SSI after instrumented spinal surgery is crucial and challenging, as it is positively associated with extended hospitalizations, increased morbidity and health care costs, and greater dissatisfaction with the initial operative procedure. There are still no universally accepted protocols for the treatment of late-onset deep SSI. In the present study, a timely diagnosis, aggressive and meticulous debridement, and the routine use of antibacterial agents are keys to successfully resolving the infections and retaining the implants. The limitations of this study are that it is a retrospective survey, the number of patients is small, and the follow-up period is short. Nevertheless, this study provides some reference for the treatment of late-onset deep SSI after instrumented spinal surgery.

## Abbreviations

SSI: Surgical site infection
MRI: Magnetic resonance imaging
ESR: Erythrocyte sedimentation rate
CRP: C-reactive protein
PCT: Procalcitonin
TLIF: Transforaminal lumbar interbody fusion
PLIF: Posterior lumbar interbody fusion
ASA: American Society of Anesthesiologists
ESBL: Extended-spectrum ß-lactamase
MRSA: Methicillin-resistant *Staphylococcus aureus*
CSD: Closed-suction drain
HVWD: High vacuum wound drainage system
VAC: Vacuum-assisted closure
